# *Leishmania amazonensis* Promastigotes Present Two Distinct Modes of Nucleus and Kinetoplast Segregation during Cell Cycle

**DOI:** 10.1371/journal.pone.0081397

**Published:** 2013-11-21

**Authors:** Marcelo Santos da Silva, Jomar Patrício Monteiro, Vinícius Santana Nunes, Elton José Vasconcelos, Arina Marina Perez, Lúcio de Holanda Freitas-Júnior, Maria Carolina Elias, Maria Isabel Nogueira Cano

**Affiliations:** 1 Departamento de Genética, Instituto de Biociências, Universidade Estadual Paulista (UNESP), Botucatu, São Paulo, Brazil; 2 Universidade Estadual de Campinas (UNICAMP), Campinas, São Paulo, Brazil; 3 Empresa Brasileira de Pesquisa Agropecuária (Embrapa) Caprinos e Ovinos, Sobral, Ceará, Brazil; 4 Seattle Biomedical Research Institute, Seattle, Washington, United States of America; 5 Laboratório Nacional de Biociências (LNBio), Centro Nacional de Pesquisa em Energia e Materiais, Campinas, São Paulo, Brazil; 6 Center of Toxins, Immune Response and Cell Signaling (CeTICS), Instituto Butantan, São Paulo, São Paulo, Brazil; University of São Paulo, Brazil

## Abstract

Here, we show the morphological events associated with organelle segregation and their timing in the cell cycle of a reference strain of *Leishmania* (L.) *amazonensis* promastigotes, the main causative agent of Tegumentary leishmaniasis in the Americas. We show evidences that during the cell cycle, L. amazonensis promastigotes present two distinct modes of nucleus and kinetoplast segregation, which occur in different temporal order in different proportions of cells. We used DAPI-staining and EdU-labeling to monitor the segregation of DNA-containing organelles and DNA replication in wild-type parasites. The emergence of a new flagellum was observed using a specific monoclonal antibody. The results show that *L. amazonensis* cell cycle division is peculiar, with 65% of the dividing cells duplicating the kinetoplast before the nucleus, and the remaining 35% doing the opposite or duplicating both organelles concomitantly. In both cases, the new flagellum appeared during S to G2 phase in 1N1K cells and thus before the segregation of both DNA-containing organelles; however, we could not determine the exact timing of flagellar synthesis. Most of these results were confirmed by the synchronization of parasites using hydroxyurea. Altogether, our data show that during the cell cycle of *L. amazonensis* promastigotes, similarly to *L. donovani*, the segregation of nucleus and kinetoplast do not follow a specific order, especially when compared to other trypanosomatids, reinforcing the idea that this characteristic seems to be species-specific and may represent differences in cellular biology among members of the *Leishmania* genus.

## Introduction

*Leishmania amazonensis*, a trypanosomatid protozoan, is the main causative agent of Tegumentary leishmaniasis in the Americas. Leishmaniasis is a spectrum of diseases with different clinical forms that affects approximately 350 million people around the globe. Recent data indicated that the disease is endemic in 98 countries, with more than 1.6 million new cases per year and over 20,000 deaths annually [1,2]. 

There are no effective vaccines for leishmaniasis, and the few available drugs are expensive and toxic to the host. In addition, the occurrence of drug-resistant parasites requires the establishment of intensive research to better understand the cellular and molecular biology of these parasites [3,4].

*Leishmania* spp. belongs to the Trypanosomatidae family, which includes digenetic parasites with complex life cycles and different developmental forms in vertebrate and invertebrate hosts. This peculiarity is central to successful parasite adaptation and the movement of these parasites between vector and host. Their life cycle is characterized mainly by changes in cell shape, cell cycle, metabolism, surface coat, DNA replication and gene expression that, in this case, also have their peculiarities [5-9].

These protozoa contain a unique mitochondrion that has a dense kinetoplast region composed of a network of several thousand minicircles and a few dozen maxicircles, which form the kinetoplast DNA (kDNA) [10]. These protozoa also have a single flagellum connected to the kinetoplast basal body that emerges from a flagellar pocket, which is an invagination of the plasma membrane [11]. The length of the flagellum is tightly controlled throughout the life cycle of the parasite, especially in the promastigote form, where it is essential for mobility and survival inside the invertebrate host [12,13]. 

In trypanosomatids, the coordination of nuclear and kDNA replication throughout the cell cycle is dissimilar to higher eukaryotes where mitochondrial DNA replicates at any stage of the cell cycle [14,15]. Many authors have also described the existence of a pattern of segregation of the DNA-containing organelles (nucleus and kinetoplast) in the well studied *Trypanosoma brucei* [16], and also in *Trypanosoma cruzi* [17] and *L. tarentolae* [18]. In all of these protozoa cell cycle events follow this order: the flagellum is duplicated first, and the kinetoplast divides shortly before nuclear division, culminating with cytokinesis, which is also called post-mitosis. It was also shown that *L. donovani* and *L. mexicana*, were different from other trypanosomatids because they both segregate the kinetoplast after the onset of mitosis [19,20]. However, none of these studies demonstrated if these segregation patterns were fixed and thus shown by all cells within the studied populations or if there were different proportions of cells showing a more or less frequent pattern of segregation. Recently, some authors demonstrated the existence of cells within the same population of *L. donovani* [19] and *L. major* [21], showing not only different morphologies but also a non-fixed pattern of nucleus and kinetoplast segregation. For example in *L. donovani*, although the majority of cells segregate the kinetoplast after the nucleus, it was also observed that a small proportion of cells (about 20%) within the same population segregate the kinetoplast before the nucleus [19]. A deep description about the events occurring and the molecules involved in the cell cycle of *L. major* promastigotes revealed that both the timing of its cell cycle as well as the segregation of the kinetoplast, which occurs before the nucleus, are similar to that of *T. brucei*, although the authors detected the existence of a small proportion of cells showing different configurations, which were not considered in the cell cycle duration calculations [21]. Thus, it is more clear now that at least for some species of *Leishmania* [19-21], the order and timing of organelle segregation are not consensual and cannot be generalized, although the mechanisms that ensure proper organelle segregation in trypanosomatids have been extensively studied in relation to cell cycle control, including the establishment of networks of interaction between molecules [21,22] and the relationship between DNA replication and segregation of DNA-containing organelles [17,20,21]. 

The present article shows evidences that during the cell cycle, L. amazonensis promastigotes show two distinct modes of nucleus and kinetoplast segregation, which occur in different temporal order and in different proportions of cells. Our results demonstrate that similarly to *L. donovani* [19], in the studied population we find cells segregating either the kinetoplast before nucleus or cells doing the opposite, although *L. amazonensis* shows a larger proportion of cells (65%) segregating the kinetoplast before the nucleus whereas in *L. donovani* the majority of cells (80%) segregate the kinetoplast after the nucleus [19]. Additionally, the timing for both organelle segregation and flagellum emergence differs from *L. amazonensis* and its phylogenetically closer species (e.g. *L. mexicana* and *L. donovani*), although they all have a similar population doubling time (about 7h) [19,20] this article. Therefore, our results reinforce the idea that cell cycle events involving the segregation of DNA-containing organelles seems to be species-specific and may represent differences in cellular biology among members of the *Leishmania* genus. 

## Materials and Methods

### Cell growth

 A pure culture of *L. amazonensis* promastigotes (MHOM/BR/1973/M2269) were grown at 27 °C in M199 medium (Cultilab) supplemented with 10% (v/v) heat-inactivated fetal calf serum (Cultilab), 25 mM HEPES and 1% (v/v) antibiotic/antimycotic solution (Cultilab). 

### Cell cycle analysis

Formaldehyde-fixed and DAPI-stained exponentially growing promastigotes (~1,186 cells) were examined under a Nikon 80i fluorescent microscope (100x magnification) to observe the nucleus and kinetoplast and to estimate the duration of nuclear and kinetoplast mitosis/division (M/D, respectively) and post-mitosis/post-division (post-M/post-D), according to the Williams formula [23]:$x=\frac{\ln(1-y/2)}{-\alpha }$, where x is the cumulative time within the cycle until the end of the stage in question, y is the cumulative % of cells up to and including the stage in question (expressed as a fraction of one unit), and α is the specific growth rate.

To estimate the duration of S phase, we used the Woodward and Gull formula [16]:$S=\frac{1}{\alpha }\ln\left[L+e^{\alpha \left(Z\right)}\right]-\left(Z+t\right)$, where *L* is the proportion of cells exhibiting EdU-labeled nuclei, α = ln 2/T (*T* = generation time expressed in hours), Z = G2 + (M or D) + (post-M or post-D), and *t* is the duration of the EdU labeling period in hours. 

### EdU labeling

Exponentially growing promastigotes were incubated for a minimum of 1 h with the thymidine analog EdU (5-ethynyl-2'-deoxyuridine). Parasite samples remained exposed to EdU, and cell samples were taken from the culture at time zero (after 1 h of incubation) and then every 5 min until we detected cells containing two EdU-labeled nuclei, which correspond to the period between the end of the S_N_ phase and the end of mitosis. EdU incorporation was detected using click chemistry and azide labeled with Alexa Fluor 492, according to manufacturer instructions (Click-iT Edu Image kit, Invitrogen). Images were analyzed with a Nikon 80i fluorescence microscope and captured with a digital camera (DS-Fi1, Nikon).

### Indirect immunofluorescence (IIF)

EdU-labeled promastigotes cells were washed with 1X PBS (137 mM NaCl, 2.7 mM KCl, 10 mM Na_2_HPO_4_ and 2 mM KH_2_PO_4_) and fixed in 1% (v/v) formaldehyde in 1X PBS for 5 min at room temperature. Cells were then treated with 0.1% Triton-X 100 in 1X PBS for 10 min and free aldehyde molecules were neutralized with 0.1 M glycine in 1X PBS for 10 min at room temperature. Fixed cells were washed with 1X PBS and incubated with the monoclonal antibody MAbAC (culture supernatant), which recognizes an unknown conserved trypanosomatid flagellar structure [24]. This antibody was raised by immunizing BALB/c mice with insoluble detergent extracts enriched in a cytoskeletal fraction of *T. cruzi* epimastigotes [24]. In the reaction, MAbAC was diluted (1:10) in blocking solution (4% (w/v) bovine serum albumin) and was incubated for 12 h at 4 °C. Goat anti-mouse IgG labeled with Alexa Fluor 555 (Invitrogen) was used as the secondary antibody. Cells were deposited on poly-L-lysine coated slides for 15 min, and VECTASHIELD^®^ Mounting Medium with DAPI (Vector Labs) was used as the anti-fade mounting solution and to stain nuclear and kinetoplast DNA. For these experiments, images were analyzed with a Nikon 80i fluorescence microscope and captured with a digital camera (DS-Fi1, Nikon). When necessary, images were superimposed using NIS elements software (version Ar 3.10) or ImageJ software (version 1.43 u).

### Cell cycle synchronization

For HU-synchronization, parasites in mid-log phase were treated for 14 hours with 5 mM HU (Sigma) at 27 °C. Control cultures were incubated under identical conditions except that HU was absent. After HU treatment, synchronized and control parasites were harvested by centrifugation at 2,300 *g* for 5 minutes at 4 °C for the complete removal of HU, and the parasites were recovered in twice the original amount of fresh M199 supplemented medium. Samples containing approximately 2x10^6^ parasites were collected hourly for 7 hours, harvested by centrifugation, washed in 1X PBS (0.14 M NaCl, 2,7 mM KCl, 10 mM Na_2_HPO_4_, 2 mM KH_2_PO_4_, pH 7.4), fixed in 1% formaldehyde for 5 minutes at room temperature, washed again and suspended in 1X PBS. Cells were permeabilized with 0.1% Triton-X 100 and incubated with 40 μg of RNAse A (Invitrogen) for at least 2 hours at 37 °C. To measure the DNA content, the cells were stained with 40 μg/ml propidium iodide overnight (Sigma, St. Louise, MO, U.S.A.) and were analyzed using the FACS Calibur (Becton Dickinson) and CELLQuest software (BD Biosciences) for data extraction. Flow cytometry data analysis was performed using WinMDI v2.8 (http://facs.scripps.edu/software.html) to construct histograms (events x *FL2 area*), scatter plots (*FL2 area* x *FL2 width* and *SSC-Height* x *FSC-Height*) and to determine synchronization percentages.

## Results

### The segregation of nucleus and kinetoplast in *Leishmania amazonensis* promastigotes occur in different temporal order in different proportions of cells

**Figure 1:**
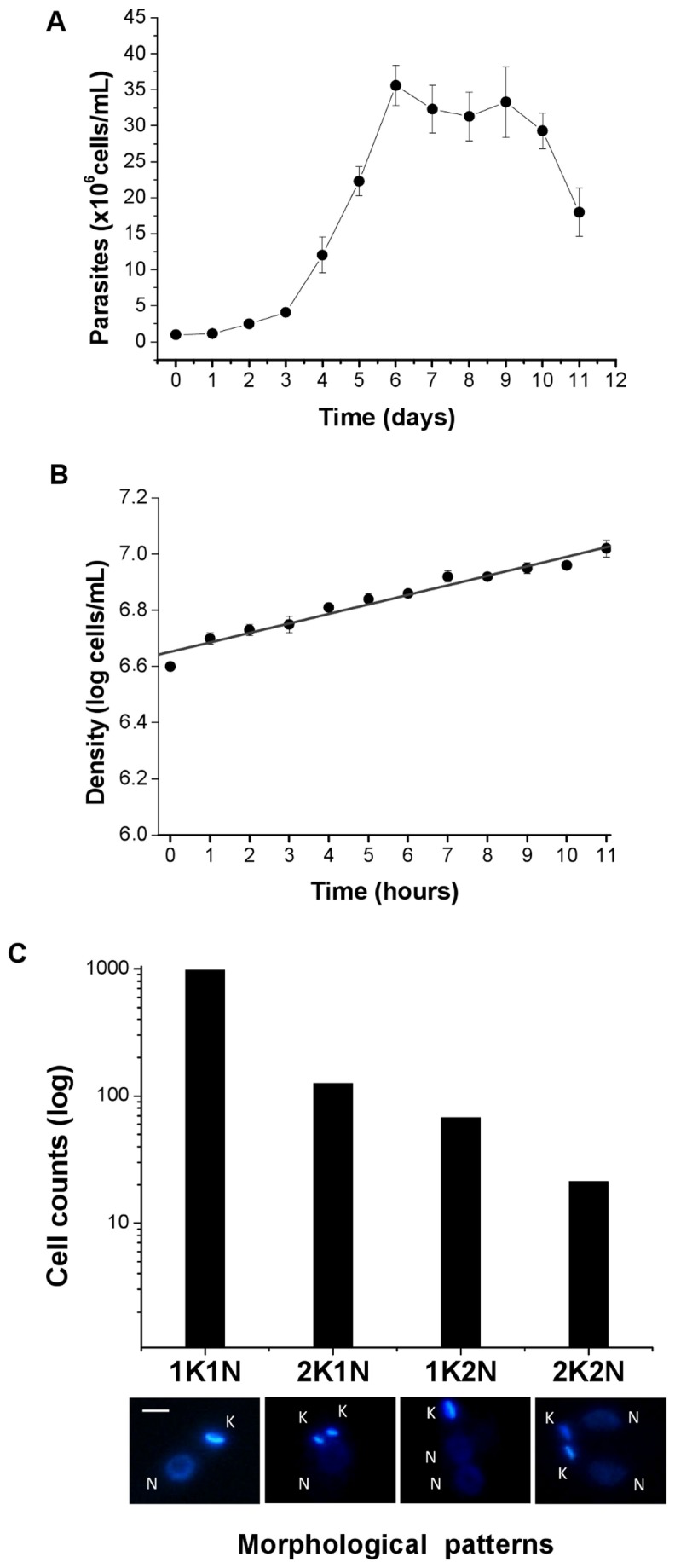
Estimation of generation time and the profile of organelle segregation in *L. amazonensis* promastigotes. Panel **A**) Typical growth curve of *L. amazonensis* promastigotes in M199 medium at 28 °C. Promastigotes grew logarithmically in the range 1 x 10^6^ to 3.5 x 10^7^ cells ml-1. Panel **B**) Cell density was measured hourly over 12 h. The generation time was calculated to be 7 h (r^2^ = 0.983). Errors bars indicate SD of three independent assays. Panel **C**) Distinct morphological patterns observed in exponentially growing *L. amazonensis* promastigote cultures. The data were obtained from 1,186 cells counted from three independent axenic cultures of wild-type *L. amazonensis* promastigotes. Images are representative of DAPI-stained cells showing different organelle segregation, 1K1N are cells not in division, 1K2N and 2K1N are cells in division, and 2K2N are cells in post-M/post-D. Images were captured using a Nikon 80i and NIS element v.3.0 Software. K = kinetoplast, N = nucleus. Bars = 2 µm.

*Leishmania amazonensis* promastigotes were cultured in M199 medium supplemented with 10% fetal bovine serum at 28 °C, and exponentially growing cells (Figure 1A) were used for all experiments described in this article. To calculate *L. amazonensis* generation time, we used an initial inoculum of 5 x 10^6^ cells.mL^-1^, and the counts were performed hourly using a Neubauer chamber (Figure 1B). Analysis of these counts resulted in an estimated generation time of 7 hours for *L. amazonensis* promastigotes (Figure 1B). This value was subsequently used to determine the duration of each cell cycle phase, with the concomitant morphological analyses shown below.

We examined 1,186 DAPI-stained wild-type *L. amazonensis* promastigotes in exponential growth and observed that most of them (973 cells) contained one kinetoplast and one nucleus (1K1N). Among the cells in division (191 cells), we observed two distinct modes of nucleus and kinetoplast segregation: one in which 125 cells, representing 65% of the 191 cells, segregated the kinetoplast before the nucleus and thus contained two kinetoplasts and one nucleus (2K1N), and the other, representing 66 cells, or the remaining 35% of the 191 cells, segregated the kinetoplast after the nucleus and had one kinetoplast and two nuclei (1K2N) (Figure 1C). The remaining 22 cells were in post-Mitosis/post-kinetoplast division (post-M/post-D) and had two kinetoplasts and two nuclei (2K2N) (Figure 1C).

### The new flagellum emerges during S/G^2^ phase before the segregation of DNA-containing organelles

**Figure 2:**
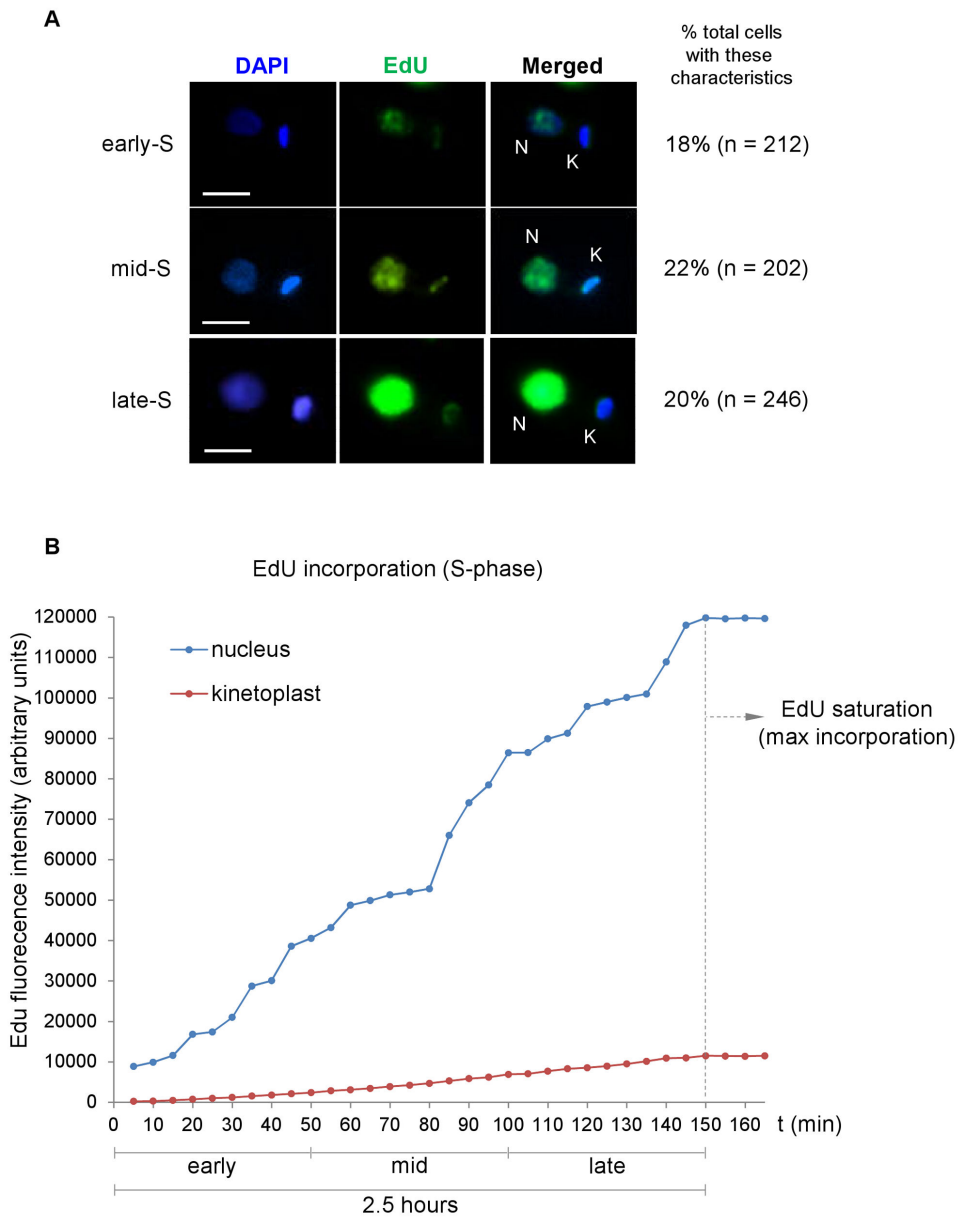
DNA replication occurs simultaneously in the nucleus and in the kinetoplast of *L. amazonensis* promastigotes. Panel **A**) EdU incorporation was revealed by click chemistry using azide labeled with Alexa 594. DAPI was used to stain DNA in the kinetoplast (K) and in the nucleus (N). The images represent cells in early, mid and late S phase, determined by EdU incorporation and the morphology of DAPI-stained nucleus and kinetoplast. The column on the right shows the percentage of cells in the population that had the same characteristics. Bars, 2 µm. Panel **B**) The amount of EdU-incorporation was estimated by fluorescence intensity using NIS elements v.3.0 software. The duration of S phase for each organelle was determined by morphology and increased fluorescence intensity, which was directly proportional to increased EdU incorporation. The maximum fluorescence intensity/incorporation (signaled as EdU saturation) indicated the end of S-phase for each DNA-containing organelle (approximately 120,000 a.u for nuclear DNA and approximately 10,000 a.u for kinetoplast DNA).

To estimate the timing of DNA replication in the nucleus and in the kinetoplast, *L. amazonensis* promastigotes were incubated with EdU (5-ethynyl-2'-deoxyuridine), and cell samples were observed every 5 minutes until the cells showed the strongest EdU fluorescent signals in both DNA-containing organelles (nucleus and kinetoplast). The images shown in Figure 2A are representative of cells in different stages of S phase (early, mid and late) and were chosen based mainly on morphology of DAPI-stained nucleus and kinetoplast and the increased fluorescence intensity of both EdU-labeled organelles, as estimated using NIS software (Figure 2B). This helped us estimate S phase duration as approximately 2.5 h, which agreed with the value obtained using the Woodward and Gull formula (see below) [16]. The results also showed that DNA replication in *L. amazonensis* promastigotes is an event that most likely occurs simultaneously in the nucleus and in the kinetoplast. 

**Figure 3:**
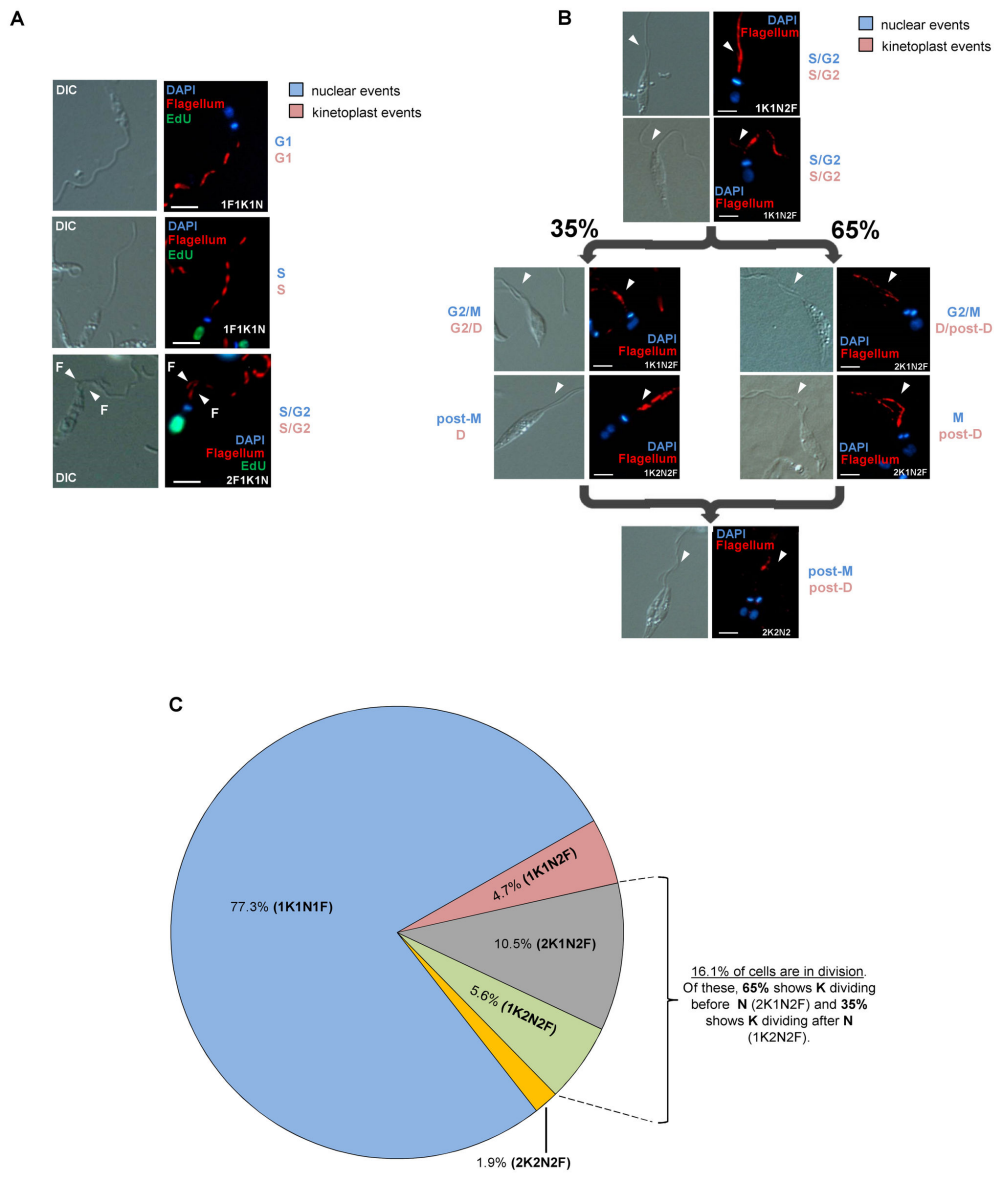
Morphological patterns and flagellum emergence during the cell cycle of *L. amazonensis* promastigotes. Panel **A**) The panel shows that a new flagellum emerges in S/G^2^ phases of 1K1N cells before the segregation of both kinetoplast and nucleus. EdU label was used as mark of DNA replication in both DNA-containing organelles. Panel **B**) The panel shows nuclear (blue) and kinetoplast (pink) events and demonstrates differences in the percentage of cells that segregate the kinetoplast before and after the nucleus. We can also observe that organelle segregation takes place after the appearance of a new flagellum in S and G2 phases of both organelles. In panels A and B, nuclear (blue) and kinetoplast (pink) events are, respectively labeled as G1, S, G2, M, D (kinetoplast division) and Post-M/Post-D (or cytokinesis). DIC (differential interference contrast), DAPI staining (blue), EdU labeling (green) and flagellum labeling with monoclonal antibody MAbAC (red). N = nucleus, K = kinetoplast. Bars = 2 µm. Panel **C**) The diagram represents a summary of the results shown in A and B.

**Figure 4:**
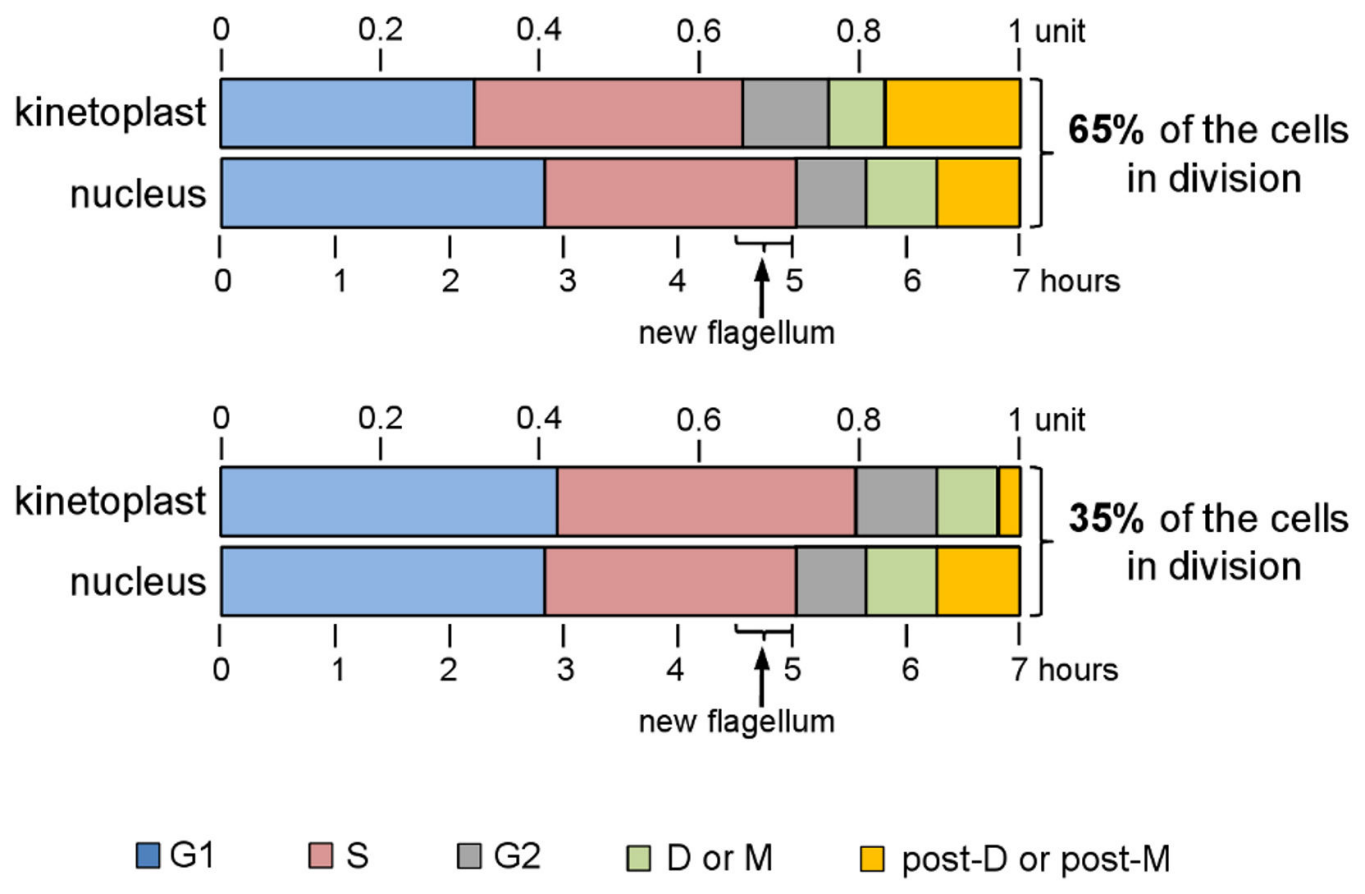
Cell cycle periods and timings for *Leishmania amazonensis* promastigotes. Summary of the calculated durations and sequences of the nuclear and kinetoplast events are separately represented for 65% of cells that divide the kinetoplast before the nucleus (top) and for the remaining 35% that do the opposite (bottom). The estimated timing for the appearance of a new flagellum is also noted. These calculations were based on the generation time for *L. amazonensis* promastigotes (7 h), which corresponds to one unit of the cell cycle.

To estimate the timing for the emergence of a new flagellum from the cell body of *L. amazonensis* promastigotes, parasite cells were incubated with EdU followed by flagellum labeling with MAbAC (culture supernatant) [24]. For this experiment, parasites remained exposed to EdU, and cell samples were taken every 5 min followed by flagellum labeling. The collected images shown in Figure 3A are representative of *L. amazonensis* promastigotes in G1, S and S/G^2^ cell cycle phases for nucleus and kinetoplast and were selected based on the morphology of DAPI-stained and EdU-labeled organelles and flagellum appearance using an indirect immunofluorescence (IIF) assay. We observed that the new flagellum protrudes during S/G^2^ phase in 1N1K cells and thus before the segregation of both DNA-containing organelles as shown in Figure 3B and in other *Leishmania* species [20,21]. Although we could not determine the exact timing of flagellum appearance, not even if it emerged during S_N_/G2_N_ or during S_K_/G2_K_, there is an estimation in Figure 4 based on the timing calculated for each kinetoplast and nuclear cell cycle event, which shows that it takes approximately 4.5 h (0.64 units of the cell cycle) within the 7 h cell cycle for a new flagellum to emerge from *L. amazonensis* promastigotes. 

In summary, after examining 1,186 DAPI-stained cells, we observed that 191 cells (16.1% of the total) were in division (segregating one or both nucleus and kinetoplast) and had two flagella. Among these 191 cells, 65% (125 cells or 10.5% of the total) were 2K1N2F and 35% (66 cells or 5.6% of the total) were 1K2N2F, meaning that these cells did not end the cytokinesis, but they segregated the kinetoplast (D cells in Figures 3B and 3C) or the nuclei (M cells in Figures 3B and 3C). From the total of 1,186 cells, only 1.9% (22 cells) were in cytokinesis (post-M/post-D cells in Figures 3B and 3C). Thus, among the exponentially growing promastigotes of *L. amazonensis* we can observe cells with the same morphologies presented by both *L. mexicana* and *L. major* (1K1N1F, 1K1N2F, 2K1N2F, 1K2N2F, 2K2N2F) [20,21], although *L. amazonensis* differs from them in terms of the timing and proportion of cells that segregate the kinetoplast before or after the nucleus. The diagram shown in Figure 3C summarizes our findings and shows the proportions of these cell configurations in the studied population.

### Estimation of cell cycle event timing in *L. amazonensis* promastigotes shows that DNA replication seems to be coordinated with the segregation of both DNA-containing organelles

To calculate the duration of M and post-M nuclear events, we used the values estimated for the population doubling time (Figure 1B) and for the proportion of cells within the 1,186 cells that have distinct N/K configurations, including the order and timing estimated for organelle segregation (Figures 1C and 3C). 

The sum of cells with 1K2N2F (66 cells or 5.6% of the total) and 2K2N2F (22 cells or 1.9% of the total) configurations was used to estimate the duration of post-M phase, which was estimated to be 0.72 h or 0.1 units of the cell cycle using the Williams formula [23]. To estimate the duration of M phase, we used cells with the 2K1N2F configuration (125 cells or 10.5% of the total), which represented cells that did not reach the end of mitosis and thus had presented the nucleus in division but not yet segregated; additionally, cells with this configuration were considered mitotic by Ambit et al. (2011) [21]. Using the Williams formula [23], the duration of M phase was estimated to be 0.62 h or 0.09 units of the cell cycle (Table 1, Figures 3B, 3C and 4). 

**Table 1:** Timing of organellar events.

**Nuclear events**				
**Stage**	**Proportion of cell cycle**		**Hours**	
G1_N_	0.40		2.83	
S_N_	0.32		2.20	
G2_N_	0.09		0.63	
M	0.09		0.62	
post-M	0.10		0.72	
**Kinetoplast events**				
**Stage**	**Proportion of cell cycle**		**Hours**	
	**65 % of cells**	**35 % of cells**	**65 % of cells**	**35 % of cells**
G1_K_	0.32	0.42	2.22	2.95
S_K_	0.33	0.37	2.35	2.61
G2_K_	0.11	0.10	0.76	0.72
D	0.07	0.08	0.49	0.53
post-D	0.17	0.03	1.18	0.19

To estimate the duration of nuclear G2 phase (G2_N_), *L. amazonensis* promastigotes were maintained in the presence of EdU until cells containing two EdU-labeled nuclei were observed (data not shown). The results showed that 1K2N or 2K2N cells were first detected 1.25 h after EdU incorporation, meaning that cells in the end of S phase took 1.25 h to go through G2 phase and mitosis. Considering this finding and that the duration of M phase in *L. amazonensis* promastigotes is 0.62 h, we could estimate the duration of G2_N_ as 0.63 h (0.09 units of the cell cycle) (Table 1 and Figure 4). 

Using the values obtained for post-M, M and G2_N_ and the proportion of cells that exhibited EdU-labeled nuclei after 1.25 h of EdU exposure (50% of cells), we were able to calculate the duration of S_N_ phase; according to the Woodward and Gull formula [16] this was estimated to be 2.2 h or 0.32 units of the cell cycle. This value is similar to the time estimated for the duration of DNA replication in both DNA-containing organelles (2.5 h) using the fluorescence intensity of EdU-labeled cells (Figure 2B).

Finally, the duration of G1_N_ phase was estimated to be 2.83 h (0.4 units of the cell cycle), calculated by taking the difference between the sums of the timings estimated for the other phases (S_N_+G2_N_+M+post-M) and the generation time (Table 1 and Figure 4). Therefore, in a 7 h cell cycle, the longest nuclear event was G1_N_ phase followed by S_N_ (2.2 h), post-M (0.72 h), G2_N_ (0.63 h) and M (0.62 h). 

Using the same approaches described above, we could also estimate the timing of each cell cycle phase for the kinetoplast events. For this calculation, we also used that group of 191 cells from the total of 1,186 DAPI-stained cells, which were in division and a step before cytokinesis (Figure 3C). Within this group, we could observe that 65% of cells (125 cells) segregate the kinetoplast before the nucleus (2K1N), and the remaining 35% of the cells (66 cells) segregate the kinetoplast after the nucleus (1K2N) (Figures 3B and 3C). Summarizing our estimations, according to the percentage of cells in D or post-D and the population doubling time, the duration of both phases for 65% of cells that segregate the kinetoplast before the nucleus were 0.49 h (0.07 units of the cell cycle) and 1.18 h (0.17 units of the cell cycle), respectively, and for the 35% that do the opposite, durations were 0.53 h (0.08 units of the cell cycle) and 0.19 h (0.03 units of the cell cycle), respectively (Table 1 and Figure 4). 

Based on the observations of cells containing two EdU-labeled kinetoplasts, as detected 1.25 h after the end of S_k_ phase, the duration of G2_k_ for 65% of cells was estimated to be 0.76 h (0.11 units of the cell cycle) and was 0.72 h (0.10 units of the cell cycle) for the other 35% of cells, which is in agreement with the late kinetoplast segregation observed within this group of cells (Table 1 and Figures 3B and 4). We then used the Woodward and Gull formula [16] to estimate the duration of S_k_ as 2.35 h for 65% of cells (0.33 units of the cell cycle) and 2.61 h for 35% (0.37 units of the cell cycle) (Table 1 and Figure 4). The values obtained are also similar to the time estimated for kinetoplast DNA replication (2.5 h) using EdU fluorescence intensity (Figure 2B). Finally, the time estimated for G1_k_ phase for 65% of cells was 2.2 h (0.32 units of the cell cycle) and was 2.95 h (0.42 units of the cell cycle) for 35% of cells; these times were also calculated using the difference between the sums of the timings estimated for the other phases (S_k_+G2_k_+D+post-D) and the generation time (Table 1 and Figure 4). Therefore, the greatest discrepancies among kinetoplast events in both cell configurations (2K1N and 2K2N) occurred in G1_k_ and post-D phases (Table 1 and Figure 4). 

Altogether, the results indicate that in *L. amazonensis* promastigotes, the segregation of nucleus and kinetoplast parallels with DNA replication in both organelles, although segregation of these organelles occurs in a distinct temporal order in different proportions of cells within the population.

### HU-synchronized parasites have the same organelle segregation pattern observed in non-synchronized wild-type parasites

**Figure 5:**
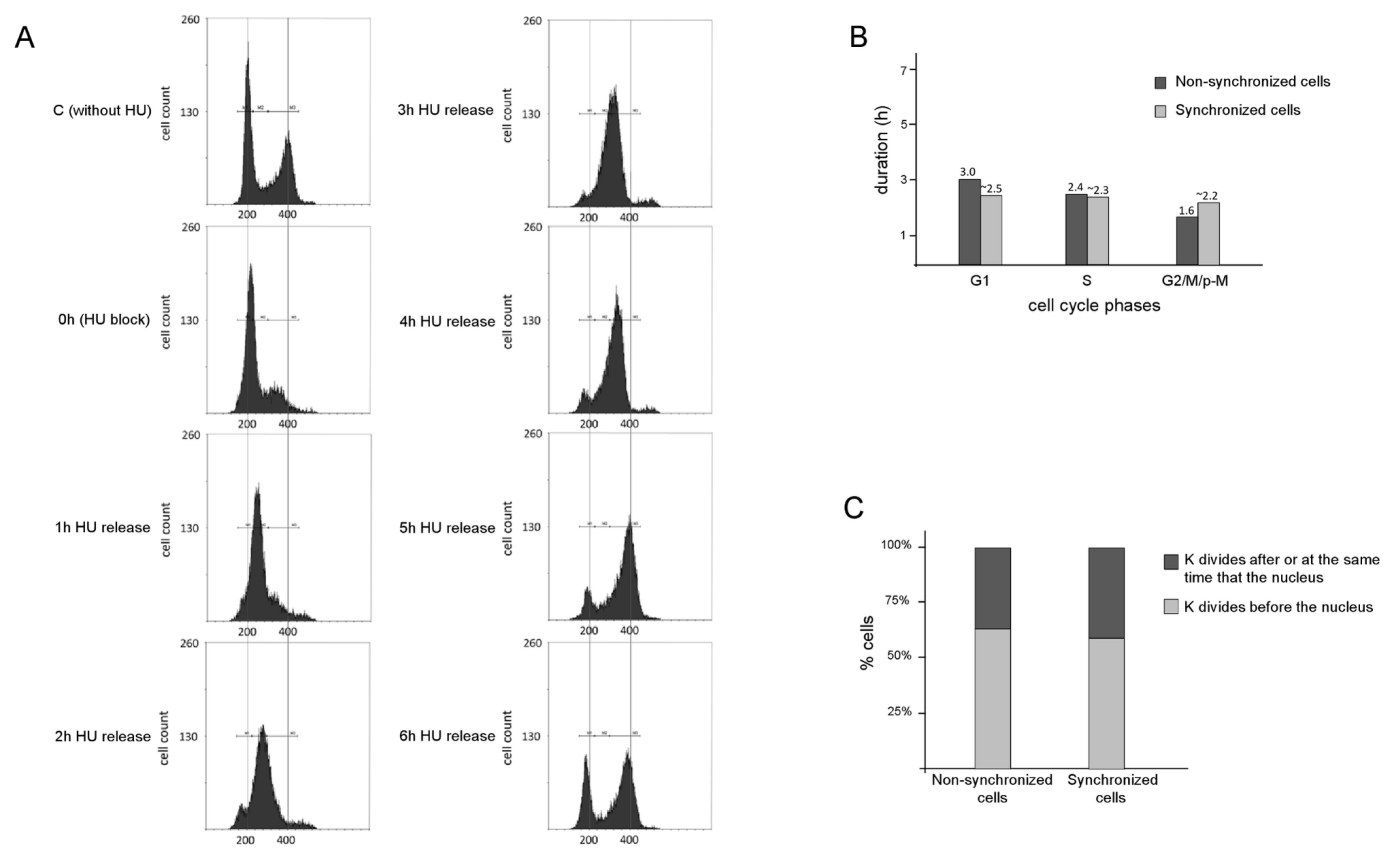
HU-synchronized *L. amazonensis* promastigotes have the same behavior of organelle segregation as wild-type parasites. Panel **A**) Histograms show DNA content per cell after propidium iodide staining and FACS analysis (20,000 total events counted per timepoint). The positions of G1, S, and G2/M, respectively, derived from the data using CellQuest software. Samples were harvested hourly after release from HU. Panel **B**) Graph shows a comparison of the timing estimated for nuclear + kinetoplast cell cycle phases (G1, S, G2/M/post-M) between non-synchronized and HU-synchronized cells. Panel **C**) Percentage (%) of HU-synchronized and of non-synchronized cells with differences in organelle segregation. In this assay we analyzed 1,186 non-synchronized cells used in most assays of this article and 1,020 synchronized cells which were harvested and analyzed (using DAPI staining) each 30 minutes after HU release up to the end of the cell cycle (around 4,5h after HU release). The percentage showed is related only to cells in division.

To determine if the morphological patterns shown by wild-type *L. amazonensis* promastigotes are reproducible after synchronizing cells in culture, we treated exponentially growing parasites with hydroxyurea (HU). DNA content was estimated by cell sorter analysis using propidium iodide (Figure 5A), and the order of organelle segregation was observed during S phase using DAPI-stained cells (data not shown).

The synchronization of the *L. amazonensis* promastigote forms (approximately 60% of the cells) allowed us to estimate the timing of the main phases of the cell cycle (G1, S and G2/M). According to cell sorter analysis (Figure 5A), the duration of one promastigote cell cycle was approximately 7 hours. Here, we were able to detect an increase in propidium iodide fluorescence per cell as early as 1 hour after release from HU arrest. Using this approach, S phase was estimated as the time that fluorescence readings increased and cells reached 4n content, indicating that most cells reached double the initial fluorescence value in the histograms due to ongoing DNA replication. By this method, DNA replication (S phase) lasted for approximately 2.5 hours, G2/M/post-M for approximately 1.5 hours and G1 for approximately 3 hours (Figures 5A and Table 2). These values are all in perfect agreement with the values obtained for the nuclear events in non-synchronized wild-type parasites (Table 1 and Figure 5B) and reinforce the reliability of the data presented here. It is noteworthy that these estimations using FACs analysis are somewhat complicated because some cells remain attached to each other after cell cycle completion [20]. It is also important to recall that the concentration of HU used in these experiments does not cause DNA damage in *L. amazonensis* promastigotes [25], maintaining the order of each cell cycle phase, with an approximated number of cells in each phase and a similar proportion of cells segregating the kinetoplast either before (approximately 60%) or after the nucleus (approximately 40%) (Figures 5B and 5C).

**Table 2:** Results of FACS analysis.

**Time**	**G1_N_ and G1_K_ (%)**	**S_N_ and S_K_ (%)**	**G2_N_/M/post-M and G2_K_/D/post-D (%)**
C (without HU)	44.44 ± 0.8	12.22 ± 0.3	43.34 ± 0.5
0h (HU block)	45.84 ± 6.2	31.36 ± 4.4	22.8 ± 2.1
1h release	24.04 ± 4.5	54.18 ± 6.3	21.78 ± 2.9
2h release	17.54 ± 8.1	52.3 ± 2.3	30.16 ± 9.5
3h release	7.07 ± 1.3	28.28 ± 5.6	64.65 ± 5.7
4h release	8.4 ± 1.7	20.42 ± 6.5	71.18 ± 7.9
5h release	11.48 ± 2.7	14.74 ± 4.3	73.78 ± 4.5
6h release	23.19 ± 5.6	10.31 ± 1.7	66.5 ± 3.9

## Discussion

The morphological events that accompany the cell cycle in trypanosomatids have been studied in detail in some species, and it appears that for some of them there was a general consensus on the order of events. In most organisms (e.g., *T. brucei*, *T. cruzi*, and *L. tarentolae*), the first event is the emergence of the new flagellum, followed by kinetoplast segregation, nuclear division and finally cytokinesis [17,18,26,27]. However, recent studies reveal peculiarities among different *Leishmania* species that require more investigation [19-21].

In the present study, DAPI staining and EdU labeling were used to observe changes in the morphology of both DNA-containing organelles and DNA replication in *L. amazonensis* promastigotes. It is worth noting that the phenomenon described here was repeatedly observed in both exponentially growing and HU-synchronized cultures even though DAPI-based analysis has considerable limitations that omit many details of the division of these organelles and is also a marker of late division, although not necessarily a good marker of mitosis or kinetoplast division initiation or completion [28]. EdU-labeling was used as marker to follow DNA replication in asynchronous cultures, and it helped us estimate the timing of the S-phase in both DNA-containing organelles by the measurement of their fluorescence intensity signal and area. We observed that the strongest fluorescent signal captured in both organelles accompanied changes in size and in organelle morphology (Table 3 and Figure 2A). For example, nuclei with the strongest fluorescent signal always had increased organellar area (Table 3), most likely indicating that they were approaching the end of S_N_ (late S phase/beginning of G2 phase). The kinetoplast morphology also changed from a small stick to a rounded morphology at late S_k_ when it also showed a strongest EdU fluorescent intensity signal (Figure 2A). Both events were accompanied by the emergence of the new flagellum from the cell body at late S phase (Figures 3A and 3B). It is also worth emphasizing that after examining ~1186 cells, the difference in the proportion of cells that have distinct behavior in organelle segregation were more evident from this point and beyond (late S phase/beginning of G2 phase) (Figures 3B and 3C). 

**Table 3:** Organellar area (µm^2^) during S phase.

**organelle**	**early-S** (n=212)	**mid-S** (n=202)	**late-S** (n=246)
nucleus	3.71 ± 0.45	4.95 ± 0.78	6.45 ± 1.02
kinetoplast	1.03 ± 0.14	1.18 ± 0.17	1.58 ± 0.24

Based on this information and the Williams [23] and Woodward and Gull [16] analyses, an interesting observation could be made. Our results show significant differences in the order and timing of *L. amazonensis* promastigote cell cycle events in comparison with other *Leishmania* species (e.g., *L. mexicana, L. major, L. donovani*, *L. infantum*) (Table 4), which somehow breaks a paradigm with respect to previous observations. 

**Table 4:** Duration of the generation time and order of kinetoplast segregation in different trypanosomatids and experimental characteristics.

**Organism**	**Generation time**	**Growth Medium**	**Temperature of growth**	**Kinetoplast segregation**	**Reference**
***L. amazonensis***	7 h	M199	27 °C	BN (65 %)	This article
				AN (35 %)	
***L. mexicana***	7.1h	M199	28 °C	AN	[20]
***L. major***	10.2 h	HOMEM	25 °C	BN	[21]
***L. donovani***	~7 h	M199	24 °C	BN (15-20 %)	[19]
				AN (80-85 %)	
***L. infantum***	6 h	RPMI 1640	26 °C	ND	[38]
***L. tarentolae***	5 h	BHI	27 °C	BN	[18]
***T. cruzi***	24 h	LIT	28 °C	BN	[17]
***T. brucei***	8.65 h	SDM-79	27 °C	BN	[16]

BN = before nucleus; AN = after nucleus; ND = not determined

Comparisons among the most closely related *Leishmania* species (e.g., *L. amazonensis* and *L. mexicana*) and the more evolutionarily divergent (e. g. *L. tarentolae*, *L. major* and *L. donovani*) show that the timing for the emergence of the new flagellum and the division of both DNA-containing organelles is markedly different [18-21], with *L. amazonensis* and *L. donovani* showing in addition, different proportion of cells displaying two modes of segregation of the DNA-containing organelles. A large proportion of *L. amazonensis* dividing cells (65%) and 20% of *L. donovani* cells, similarly to *L. major* and *L. tarentolae* segregate the kinetoplast before nucleus and a smaller proportion of *L. amazonensis* cells (35%) behave like *L. mexicana* and 80% of *L. donovani* cells, as they segregate the kinetoplast after the nucleus (Table 4). Moreover, the emergence of the new flagellum in *L. amazonensis*, *L. major* and *L. tarentolae* occurs during S/G^2^ phase and thus, prior to both nuclear and kinetoplast division [18,21] this article, whereas in *L. mexicana and L. donovani*, nuclear division and flagellum growth occur prior to kinetoplast division, with the flagellum of *L. mexicana* continuously growing even in the next generation [19,20]. 

We must also call attention to the significant differences presented, even among the closest species, in cell cycle duration and the timing of each cell cycle phase (Table 4). The duration of one cell cycle of several trypanosomatids varies, with durations of 5 hours for *L. tarentolae*, 6h for *L. infantum*, 8.65 h for *T. brucei*, 10.2 h for *L. major* up to 24 hours for *T. cruzi. L. amazonensis, L. mexicana* and *L. donovani* present similar timings for cell cycle duration (around 7 h), which might be expected as they all belong to the subgenera L. (*Leishmania*) and are considered by some authors phylogenetically close [20,29-31]. In relation to the organellar segregation, among the studied trypanosomatids shown in Table 4, only *L. amazonensis* and *L. donovani* present different proportion of cells segregating the kinetoplast either before (65% and 20%, respectively) or after (35% and 80%, respectively) the nucleus. Moreover, with the exception of *L. mexicana*, which segregates kinetoplast after the nucleus, and *L. infantum*, whose organelle segregation pattern was not determined, all other trypanosomatids, including *L. tarentolae* and *L. major*, were reported as segregating the kinetoplast before the nucleus (Table 4).

Thus, taken together all these information it is not possible to establish a relationship between the taxonomic proximity among the *Leishmania* species and the duration of cell cycle, timing for flagellum appearance and the pattern of organelle segregation. Therefore, we can conclude that in relation to the cell cycle events, each species has its own peculiarities, which may represent differences in cellular biology among members of the *Leishmania* genus. The results presented in this manuscript reinforce this hypothesis.

We speculate that these discrepancies or similarities in cell cycle duration and organelle segregation among trypanosomatids, may correspond to biological differences inherent to each species studied. For example, it was recently shown that in *T. brucei*, kinetoplast duplication and division, which occurs before nuclear segregation, is driven by cytoskeleton remodeling, with maxicircle segregation occurring as a late event during the cycle [16,32]. Thus, we hypothesize that this must be a rule among trypanosomatids, and in the case of *Leishmania* genus, the differences in organelle segregation could be due to an unknown peculiarity in the cytoskeleton remodeling machinery. 

Therefore, our observations of the morphological and chronological events during the cell cycle of *L. amazonensis* promastigotes place this species in a peculiar classification. Here, it is worth recalling that among the *Leishmania* species that are human pathogens, *L. amazonensis* is able to cause the full clinical spectra of disease manifestations, ranging from cutaneous to mucosal or visceral involvement [33]. In this study we used a *Leishmania amazonensis* WHO reference strain, MHOM/BR/1973/M2269, isolated from a patient lesion, denoting that we worked with a genetically homogenous population. It is worth mentioning that clonality in natural populations of many species of *Leishmania* isolates is a common feature [34] and reinforces that the phenomenon described here is inherent to *L. amazonensis* promastigotes. In addition, when we used HU-synchronized parasites, we confirmed the results observed in non-synchronized parasites, showing that *L. amazonensis* promastigotes behaves differently from other trypanosomatids in relation to reported patterns of organelle segregation (Figure 5). However, we know that in this case we favored nuclear events in detriment of the kinetoplast events by analyzing total DNA content by flow cytometry. But, comparison of the proportions of cells that divide the kinetoplast before and after or at the same time as the nucleus revealed no significant differences between synchronized and non-synchronized populations (Figure 5C).

Trypanosomatid cell cycle is an important field of study, as it presents many peculiarities that distinguish these parasites from their hosts, both morphologically and at the molecular level [35,36]. Furthermore, the establishment of a platform for the exploration of cell cycle-related events among pathogenic and genetically related *Leishmania* species is of high interest for the determination of potential drug targets and development of new therapies, such as the utilization of agents that impair cell division [35,37]. 

In conclusion, we characterized, for the first time, the features of the cell cycle and the timing of morphological events in *L. amazonensis* promastigotes, and the results reveal that this protozoan has its own cell cycle-related particularities that should be deeply investigated to better understand the biology of this important pathogen. 
